# Sustained Disease Control in Immune Checkpoint Blockade Responders with Microsatellite Instability-high Colorectal Cancer after Treatment Termination

**DOI:** 10.1158/2767-9764.CRC-23-0340

**Published:** 2023-12-11

**Authors:** Kristen Simmons, Jane V. Thomas, Kaysia Ludford, Jason A. Willis, Victoria S. Higbie, Kanwal P.S. Raghav, Benny Johnson, Arvind Dasari, Bryan K. Kee, Christine M. Parseghian, Michael S. Lee, Phat H. Le, Maria P. Morelli, John Paul Shen, Alisha Bent, Eduardo Vilar, Robert A. Wolff, Scott Kopetz, Michael J. Overman, Van Karlyle Morris

**Affiliations:** 1 Department of Hematology/Oncology, Baylor College of Medicine, Houston, Texas.; 2 Department of Gastrointestinal Medical Oncology, The University of Texas – MD Anderson Cancer Center, Houston, Texas.; 3 Department of General Oncology, The University of Texas – MD Anderson Cancer Center, Houston, Texas.; 4 Clinical Cancer Prevention, The University of Texas – MD Anderson Cancer Center, Houston, Texas.

## Abstract

Immune checkpoint inhibitors improve survival in patients with mismatch repair deficiency/microsatellite instability-high (MSI-H) colorectal cancer. The recurrence outcomes following discontinuation of immunotherapy after prolonged disease control have not been definitively reported in large series. Records from patients with advanced MSI-H colorectal cancer from The University of Texas – MD Anderson Cancer Center who received immunotherapy between 2014 and 2022 and stopped after prolonged clinical benefit were reviewed. Median progression-free and overall survival were estimated. Associations between the event of recurrence and coexisting mutations *(KRAS/NRAS, BRAF^V600E^*), metastatic organ involvement (lung, liver, lymph node, or peritoneum), metastatic timing (synchronous vs. metachronous), prior immunotherapy [anti-PD-(L)1 alone or in combination with anti-CTLA antibodies], etiology of MSI status (sporadic vs. hereditary non-polyposis colorectal cancer), and duration of immunotherapy were assessed. Sixty-four patients with MSI-H colorectal cancer without progression on immunotherapy were reviewed. Of these 48 and 16 received anti-PD(L)1 antibody alone or in combination with anti-CTLA-4 antibody, respectively. Median exposure to immunotherapy was 17.6 months (range, 1.3–51.9). After a median follow-up of 22.6 months (range, 0.3–71.7) after stopping immunotherapy, 56 of 64 patients (88%) remained without disease progression. Lung metastases were associated with recurrence/progression (OR, 6.1; *P* = 0.04), but coexisting mutation, primary tumor sidedness, and immunotherapy were not. These data provide a retrospective, single-institution analysis that showed that most patients with advanced MSI-H colorectal cancer do not recur after treatment cessation, regardless of the reason for stopping treatment or a variety of patient and disease features, supporting an optimistic prognosis of sustained disease control.

**Significance:**

Outcomes for patients with MSI-H colorectal cancer stopping immunotherapy after disease control remain unknown. Sixty-four patients with MSI-H colorectal cancer from our institution stopping treatment for sustained benefit or toxicity were retrospectively assessed. After median follow up of 22 months and median immunotherapy exposure of 18 months, 88% patients remained without progression. All patients who recurred or progressed and were rechallenged with immunotherapy have continued to experience disease control.

## Introduction

A mismatch repair deficiency (dMMR) or microsatellite instability-high (MSI-H) status occurs in fewer than 5% of all patients with metastatic colorectal cancer (1). MSI-H/dMMR tumor are attributed as hereditary—arising from germline mutations in one of the four mismatch repair (MMR) genes (*MLH1*, *MSH2*, *MSH6*, *PMS2*) as part of the hereditary non-polyposis colorectal cancer (HNPCC) syndrome (2, 3)—or as sporadic, arising from globally hypermethylated colorectal cancer tumors featuring loss of gene expression of *MLH1* due to promoter hypermethylation (4). Via either mechanism, loss of expression and thereby homeostatic function of subsequent MMR proteins in dMMR/MSI-H colorectal cancer tumors impairs DNA repair and results in tumors characterized by high tumor mutation burden, increased tumor neoantigens, and greater cytotoxic immune cell infiltrates within the tumor microenvironment (5–9), relative to proficient mismatch repair/microsatellite stable (MSS) colorectal cancer.

Because of increased immune recognition, an MSI-H/dMMR status is a predictive biomarker for benefit with immune checkpoint blockade with anti-programmed death-(ligand) 1 [anti-PD-(L)1] treatments not only for colorectal cancer (10) but also for other solid tumors (11–13). For patients with dMMR/MSI-H advanced, unresectable colorectal cancer, the phase III KEYNOTE-177 trial (10) demonstrated improved survival with the anti-PD-1 antibody pembrolizumab relative to standard cytotoxic chemotherapy as frontline treatment and led to an FDA approval in this setting. Approximately 50% of study participants in this trial remained without disease recurrence after 2 years. Other single-arm trials evaluating anti-PD-(L)1 antibodies as monotherapy or in combination with anti-CTLA4 antibodies have reported similar long-term disease control for patients with advanced dMMR/MSI-H colorectal cancer (14–18).

Most patients with durable benefit stop therapy after 2 years of treatment for dMMR/MSI-H colorectal cancer, although the optimal length of treatment necessary for a potentially curative outcome remains undefined. With an otherwise very favorable safety profile for immunotherapy as an oncology treatment, patients in our experience often express concern about the possibility of recurrence following cessation of these agents. To provide quantifiable data useful for clinicians for guiding such conversations, we retrospectively evaluated the University of Texas – MD Anderson Cancer Center databases to describe disease recurrence and evaluated clinical and pathologic factors associated with recurrence for patients with dMMR/MSI-H advanced colorectal cancer who had stopped immunotherapy for reasons other than disease progression.

## Material and Methods

### Patient Identification for dMMR/MSI-H Advanced Colorectal Cancer

Patients assessed were included in this retrospective review approved by the Institutional Review Board at our institution if they had received therapy between 2014 and 2022 containing at least an anti-PD-(L)1 antibody as an immunotherapy treatment. All patients had a diagnosis of advanced or metastatic adenocarcinoma of the colon or rectum that was considered to be unresectable at the time of treatment start. Documentation of a dMMR or MSI-H status was confirmed in a Clinical Laboratory Improvement Amendments (CLIA)-certified laboratory by either loss of expression of at least one MMR protein on IHC, identification of microsatellite repeats using a DNA ISH assay, or commercially available next-generation sequencing testing. Sequencing for mutations in *KRAS*, *NRAS*, and *BRAF^V600E^* were performed with a next-generation sequencing assay in a CLIA-certified laboratory. Included patients stopped immunotherapy for reasons other than disease progression (i.e., treatment benefit or unacceptable treatment-related toxicity), based upon the clinical judgment of the evaluating oncologist. Pattern of recurrence was defined as either oligometastatic or disseminated metastatic, as defined previously (19), with oligometastatic recurrence consisting of up to five metastases, three or fewer involved organs, and lack of central nervous system, peritoneal, and bone metastases. Medical records were reviewed for collection of patients’ demographic, clinical, survival, pathology, and treatment information. Written informed consent was collected from each study participant for research conduct, and all research was performed in accordance with the Declaration of Helsinki.

### Statistical Analysis

Characterization of patient and tumor information was reported using descriptive statistics. Progression-free survival (PFS) was defined as the time between date of final immunotherapy (i.e., start of surveillance) and either date of disease recurrence/progression or the date of death, whichever occurred first. Overall survival (OS) was calculated as the time between stop of immunotherapy and the date of last-follow-up or date of death, whichever occurred last. Median PFS and OS were estimated according to the Kaplan–Meier method using SPSS. A log-rank test was utilized to compare median survival between groups of interest. Associations between the event of recurrence and coexisting mutations (*KRAS/NRAS, BRAF^V600E^*), metastatic organ involvement (lung, liver, lymph nodes, or peritoneum), metastatic timing (synchronous vs. metachronous), prior immunotherapy [anti-PD-(L)1 alone or in combination with anti-CTLA-4 antibodies], etiology of MSI-H status (sporadic vs. HNPCC), and duration of immunotherapy (above vs. below the median IO duration) were assessed by Fisher exact tests, with a two-sided *P* value less than 0.05 considered significant.

### Data Availability

Data were generated by the authors but are not publicly available due to patient confidentiality and protection of private health information. Deidentified data may be provided upon reasonable request from the corresponding author.

## Results

### Patient Demographics

Among 121 patients with MSI-H/dMMR unresectable, metastatic colorectal cancer who were identified as having been treated with immunotherapy, 64 (53%) did not experience progression on immunotherapy and were included in this retrospective study (Table 1). The median age at the start of immunotherapy was 64 years (range, 27–87). Of these 64 patients, 48 (75%) received anti-PD-(L)1 therapy alone and 16 received anti-PD-(L)1 therapy in combination with anti-CTLA-4 therapy. There were 48 patients (75%) who stopped because of prolonged treatment benefit, and the remaining 16 patients (25%) had immunotherapy stopped because of toxicity. Median exposure to immunotherapy was 17.6 months (range, 1.3–51.9). Median exposure to immunotherapy for those patients who stopped because of toxicity was 7.2 months (range, 1.3–26.0).

**TABLE 1:** Patient/clinical characteristics

	Number (%)	Range
Age (years, median)	64	27–87
Gender		
Male	31 (48)	
Female	33 (52)	
Ethnicity		
Asian	3 (5)	
African American	5 (8)	
Caucasian	43 (67)	
Hispanic	13 (20)	
Primary tumor site		
Right colon	46 (72)	
Left colon	18 (28)	
Immunotherapy		
Anti-PD-(L)1 alone	48 (75)	
Combination immunotherapy	16 (25)	
Mutation status		
*KRAS/NRAS* mutation	20 (41)	
*BRAF^V600E^* mutation	15 (31)	
Wild type	14 (29)	
Etiology for MSI-H/dMMR status		
Germline mutation/Lynch syndrome	17 (36)	
Sporadic	30 (64)	
Reason for immunotherapy stop		
Disease control	48 (75)	
Toxicity	16 (25)	
Metastatic organ involvement		
Liver	12 (19)	
Lung	8 (13)	
Lymph node	39 (61)	
Peritoneum	23 (36)	
Immunotherapy duration (months, median)	17.6	1.3–51.9

### Survival Outcomes Following Cessation of Immunotherapy for dMMR/MSI-H Colorectal Cancer

**FIGURE 1:**
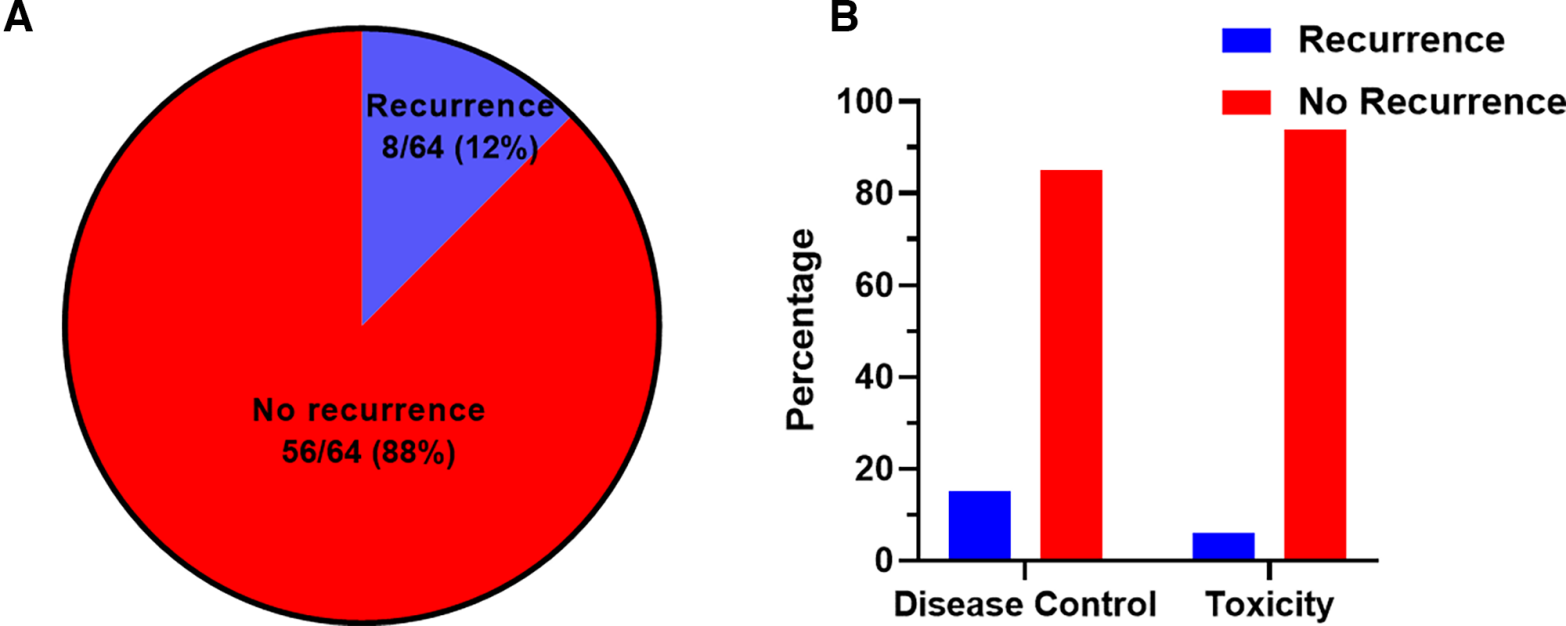
Proportion of patients with disease recurrence (**A**) and percentage of patients with disease recurrence stratified by reason for immunotherapy cessation (**B**).

After a median follow-up of 22.6 months (range, 0.3–71.7) after stopping immunotherapy, 56/64 patients (88%) remained without evidence of disease recurrence (Fig. 1A). For the 8 patients with disease recurrence, 1 had immunotherapy stopped because of prior toxicity, while 7 had no prior immunotherapy toxicity but rather stopped immunotherapy due to prolonged treatment benefit according to the discretion of the evaluating clinician. There was no statistically significant association between reason for immunotherapy cessation (disease control vs. toxicity) and subsequent disease recurrence (16% vs. 6%, respectively; *P* = 0.40; Fig. 1B). The patient who discontinued immunotherapy after only 1.3 months due to toxicity demonstrated a reduction (though not disappearance) of disease and has remained without recurrence or progression off treatment 14 months later.

There were 8 patients who did not experience sustained disease control—5 with recurrence at the same sites as previous disease and 3 with “progression” (appearance of new lesions not previously present; lung, distant lymph nodes, peritoneum; *N* = 1 each). The median time to recurrence/progression was 10.4 months (range, 0.7–19.1).

At the time of immunotherapy discontinuation, there were 28 patients (44%) who had experienced a complete response (CR) radiographically, and 36 (56%) who had stable disease or some shrinkage relative to baseline. Only 1 patient in this CR subgroup experienced recurrence, whereas 7 in the stable disease/partially responding subgroup recurred or progressed. A trend toward disease recurrence or progressing was observed for those without a CR when stopping immunotherapy [OR, 6.5; 95% confidence interval (CI): 0.75–57; *P* = 0.09].

Seven of these 8 patients were retreated with immunotherapy after recurrence or progression—4 with an anti-PD-1 antibody alone and 3 in combination with an anti-CTLA-4 antibody (Table 2). The remaining patient was not offered anti-PD-1 therapy again due to safety concerns per the discretion of the evaluating physician. None of the patients experienced worsening of disease upon restarting their immunotherapy, and have continued to tolerate treatment well without clinically significant toxicity that would warrant drug cessation.

**TABLE 2:** Characteristics of postimmunotherapy recurrence/progression

Patient	Time on immunotherapy (months)	Reason for immunotherapy stop	Pattern of colorectal cancer recurrence/progression	Restart immunotherapy?	Agent	Recurrence or progression on rechallenge?
1	23.5	Disease control	Recurrence (lymph nodes)	Yes	Nivolumab + ipilimumab	No
2	30.0	Disease control	Recurrence (lungs)	Yes	Pembrolizumab	No
3	19.3	Disease control	Recurrence (lungs)	Yes	Pembrolizumab	No
4	22.3	Disease control	Recurrence (lymph nodes)	Yes	Nivolumab + ipilimumab	No
5	35.3	Toxicity	Recurrence (peritoneum)	No	Not applicable	Not applicable
6	11.6	Disease control	Progression (peritoneum)	Yes	Nivolumab	No
7	22.7	Disease control	Progression (lungs)	Yes	Nivolumab + ipilimumab	No
8	23.5	Disease control	Progression (lymph nodes)	Yes	Nivolumab	No

**FIGURE 2:**
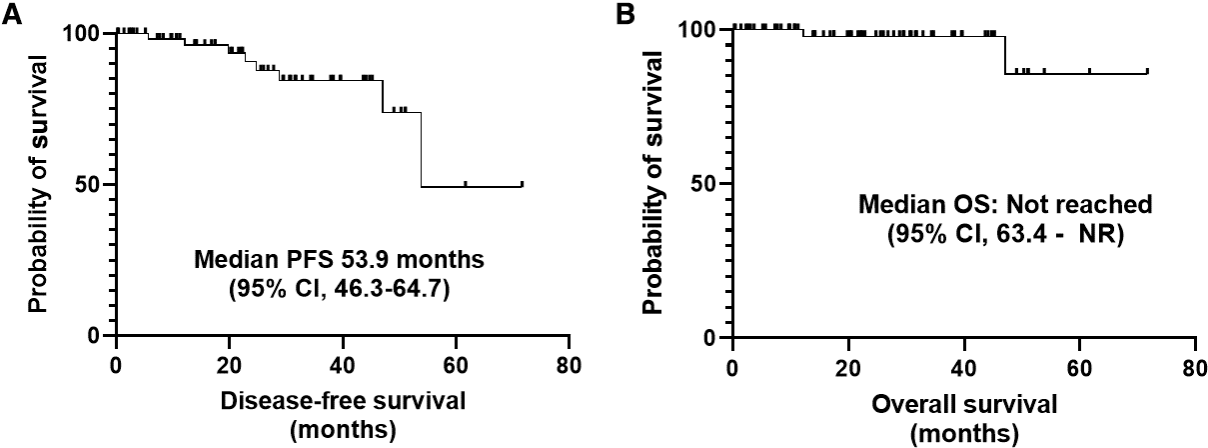
PFS (**A**) and OS (**B**) curves.

The median PFS for the entire cohort was 53.9 months (95% CI: 46.3–64.7), with estimated 1- year, 2-year, and 3-year PFS probabilities of 98%, 91%, and 84%, respectively (Fig. 2A). Median OS from the time that immunotherapy was stopped was not reached (95% CI: 63.4–NR; Fig. 2B). Only 2 patients died, of which one death was due to non–cancer-related medical condition.

### Clinicopathologic Characteristics Associated with Recurrence

**FIGURE 3:**
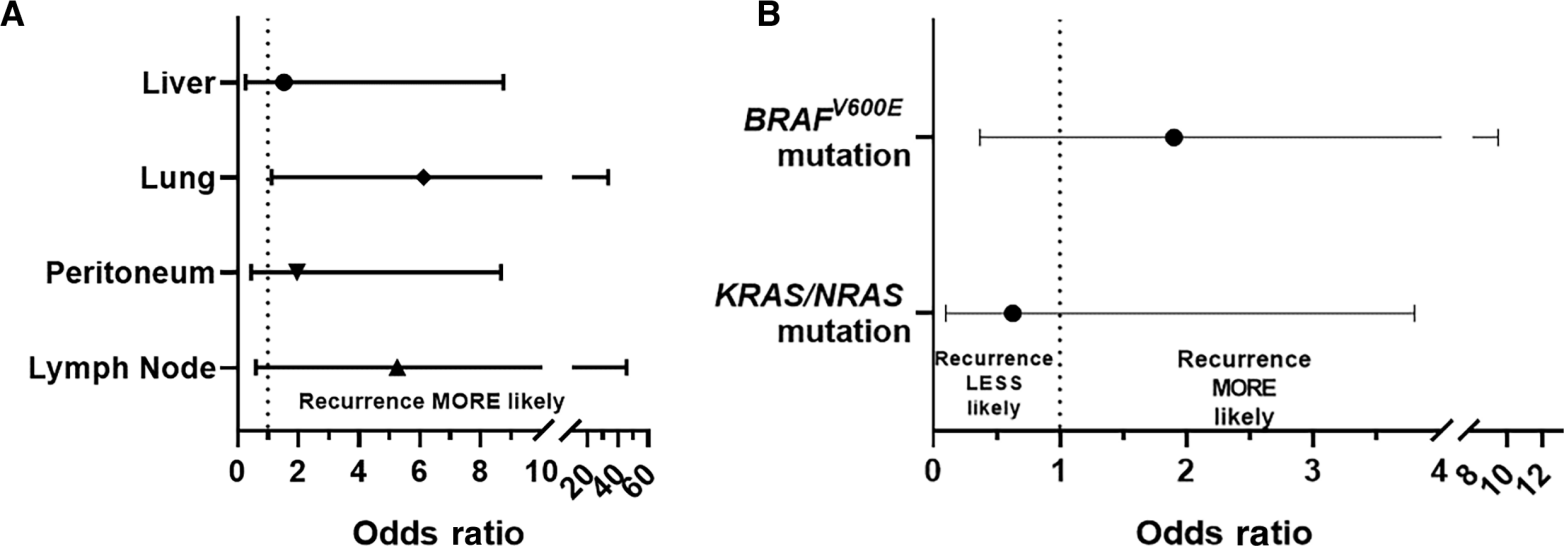
Association between disease recurrence after immunotherapy cessation and metastatic organ involvement (**A**) and mutation status (**B**).

There was no observed association between recurrence after immunotherapy cessation and metastatic organ involvement (Fig. 3A) for the liver [OR, 1.5; 95% CI: 0.3–8.7; *P* = 0.63], peritoneum (OR, 1.9; 95% CI: 0.4–8.7; *P* = 0.38), and distant lymph nodes (OR, 5.3; 95% CI: 0.6–45.6; *P* = 0.13). Increased recurrence following discontinuation of immunotherapy was observed for patients with lung metastases relative to those without lung metastases (38% vs. 9%, respectively; OR, 6.1; 95% CI: 1.1–33.5; *P* = 0.04). As seen in Fig. 3B, recurrence after immunotherapy was not associated with a *KRAS/NRAS* mutation (OR, 0.6; 95% CI: 0.1–3.8; *P* = 0.61) or *BRAF^V600E^* mutation (OR, 1.9; 95% CI: 0.4–9.5; *P* = 0.45). After recurrence/progression, molecular profiling was not performed in the majority (6/8) of cases because they were predominantly started back on immunotherapy according to the MSI-H/dMMR biomarker status. For the 2 patients who did undergo repeat testing of the circulating tumor DNA, no new acquired mutations were demonstrated at the time of recurrence compared with their baseline mutation profiling results.

**FIGURE 4:**
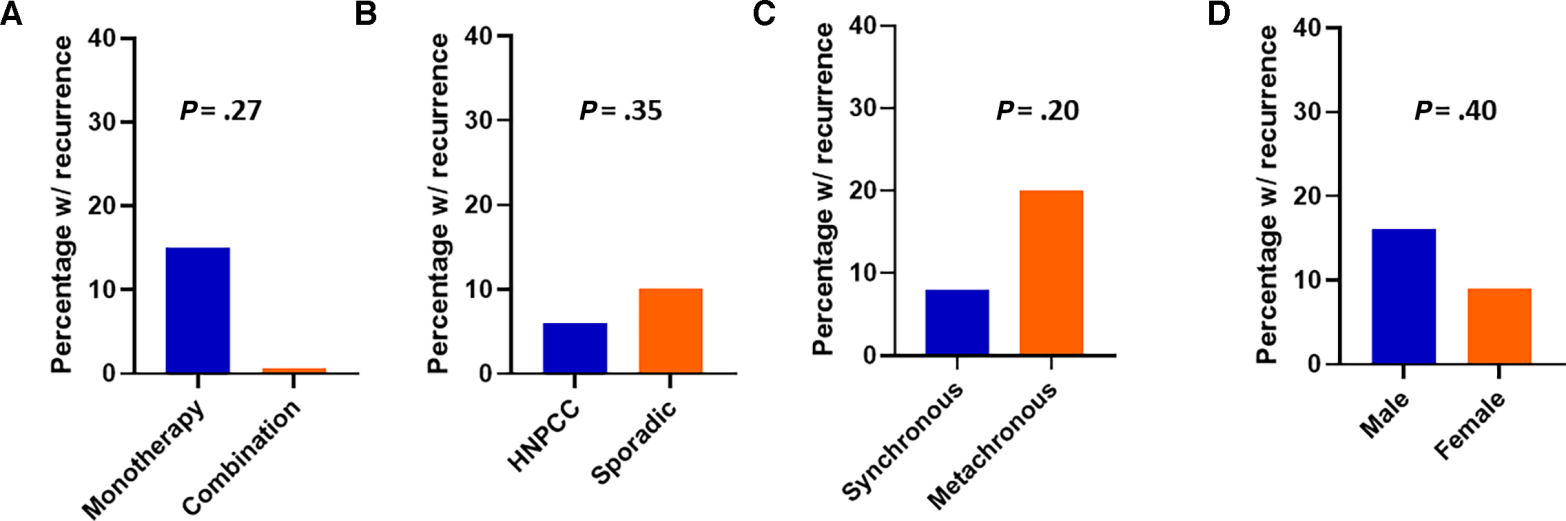
Percentage of patients with recurrence stratified by therapy regimen (**A**), etiology of MSI-H status (**B**), timing of metastatic onset (**C**), and gender (**D**).

Immunotherapy as a single agent or as a combination treatment did not affect the likelihood for recurrence after treatment discontinuation in our study. For example, 15% of patients treated with anti-PD(L)-1 therapy alone had recurrence, compared with 0% of patients treated with combination anti-PD(L)-1/anti-CTLA4 therapy (OR, 5.2; 95% CI: 0.3–97.6; *P* = 0.27; Fig. 4A). Six percent of patients with HNPCC had recurrence, compared with 13% of patients with sporadic etiology of MSI-H status (*P* = 0. 35; Fig. 4B). Timing of metastatic onset relative to the initial presentation for colorectal cancer (Fig. 4C) did not appear relevant in forecasting recurrence (8% for synchronous vs. 20% for metachronous, OR, 0.4; 95% CI: 0.1–1.7; *P* = 0.20). Sixteen percent of male patients had recurrence, compared with 9% of female patients (OR 1.9; 95% CI: 0.4–8.8; *P* = 0.40; Fig. 4D).

### Survival Outcomes Based on Attribution for Immunotherapy Cessation

We compared outcomes among the patients who stopped immunotherapy due to disease control (*N* = 48) and toxicity (*N* = 16). No difference in median PFS was observed for the group who stopped because of disease control relative to those who stopped because of immunotherapy-related toxicity [53.9 months vs. NR; HR 3.8 (95% CI: 0.88–16), Supplementary Figure].

## Discussion

Our data provide a retrospective, single-institution analysis that showed that nearly 90% patients with metastatic or unresectable MSI-H/dMMR colorectal cancer who did not experience initial progression on immunotherapy do not recur after treatment cessation. Reassuringly, we found that most patients with advanced MSI-H colorectal cancer who experienced initial clinical benefit on immunotherapy overall did well after cessation regardless of a variety of disease and patient factors, including clinical features that are generally considered prognostically unfavorable, such as mutations in *KRAS/NRAS* (20) and *BRAF^V600E^* (21–23) and metastases to the peritoneum (24, 25). This favorable effect was likewise observed regardless of the reason for stopping treatment (i.e., disease control or unacceptable toxicity from immunotherapy). Notably, for the 8 patients who did recur or progress after initially discontinuing immunotherapy, all 7 patients who were retreated with immune checkpoint blockade have continued to experience sustained disease control. While clinical trials have described the durable benefit of immunotherapy for MSI-H colorectal cancer from the start of treatment and who continue to remain on treatment, we provide here needed information regarding patient outcomes after stopping treatment.

Prior prospective clinical trials for treatment of advanced or unresectable dMMR/MSI-H colorectal cancer, such as KEYNOTE-177, have investigated outcomes in MSI-H colorectal cancer regardless of demonstrated response to immunotherapy. In such studies, those who ultimately did not clinically benefit were also included in their analysis. We sought to focus exclusively on patients with metastatic and/or unresectable MSI-H colorectal cancer that had immunotherapy stopped for reasons other than progression of disease, such as toxicity and prolonged disease control. By including only those that satisfied these criteria yet later experienced disease recurrence, we sought to identify features that correlated with disease recurrence (or alternatively, with durable/sustained benefit while off therapy). Our goal was to provide information useful to clinicians and patients with MSI-H advanced colorectal cancer for stopping immunotherapy electively.

A *post hoc* analysis of the KEYNOTE-177 trial showed that a *KRAS* or *NRAS* mutation was not predictive to benefit with pembrolizumab (10). By extension, it is reasonable to surmise that patients with dMMR/MSI-H metastatic colorectal cancer harboring a *KRAS* or *NRAS* mutation may be more likely to recur if immunotherapy is stopped. In addition, a *BRAF^V600E^* mutation is a poor prognostic biomarker for patients with colorectal cancer, regardless of the MSS status. In our series, we found that mutations in either *KRAS/NRAS* or *BRAF^V600E^* were not associated with disease recurrence in patients stopping immunotherapy for reasons other than disease progression. On the basis of these findings, it does not appear that these mutations may impact favorable outcomes in patients for whom immunotherapy is stopped electively, and these findings warrant confirmation in larger studies.

Of note, the majority of patients did not achieve a complete radiographic response at the time of their initial immunotherapy discontinuation, yet most patients have continued to remain without evidence of disease recurrence or progression. There is precedent that immune checkpoint blockade may sterilize MSI-H/dMMR tumors and eradicate all tumor despite the presence of persistent lesions noted radiographically. In support of this, a recent study showed that 12 out of 13 patients with dMMR colorectal cancer who had stable radiographic disease after immunotherapy prior to surgical resection had pathologic CR (26). Therefore, we believe that patients may stop immunotherapy after an upfront period of prolonged disease control, even if a complete radiographic response has not been achieved, as the majority of these patients will continue to experience clinically favorable outcomes without evidence of disease recurrence or progression.

Recent data suggest that site of distant metastasis may affect the response to immunotherapy for patients with colorectal cancer, likely due to differences in the tumor microenvironment. For example, a trial of anti-PD-1 and anti-CTLA4 therapies for patients with MSS, metastatic colorectal cancer reported improved response rates for patients without liver metastases than for patients with liver metastases (57% vs. 0%, respectively; ref. 27). In a single-institution retrospective analysis of 41 patients with MSI-H/dMMR metastatic colorectal cancer, treatment with pembrolizumab fared worse if patients had liver metastases than if they had non-liver metastatic colorectal cancer (28). Our retrospective cohort here was different than these analyses in excluding patients who had progressed and therefore did not benefit from immunotherapy. Indeed, only 19% of patients analyzed had liver metastases, far lower than the general population of patients with metastatic colorectal cancer, for which the prevalence exceeds 50% (29, 30). In our analysis, when we stratified by site of disease metastasis, we found no association of disease recurrence with metastases to the liver. Interestingly, a possible association between lung metastases and disease recurrence/progression was observed and merits further investigation in future studies.

We acknowledge the limitation that our data arise from retrospective study performed at only a single, large-volume, academic referral center. Validation of these findings across a population-based dataset could further generalize our findings. While a dMMR/MSI-H status is a favorable biomarker that predicts benefit to immunotherapy for patients with advanced cancers, as many as 40% of patients with colorectal cancer in this setting do not experience disease control and develop disease progression through such treatments. Recurrence is always a concern for patients and oncologists following treatment cessation.

## Conclusions

Our data reinforce the favorable outcomes starting at the time of immunotherapy discontinuation, with nearly 90% of patients achieving sustained disease control off treatment. Favorable outcomes were observed regardless for the clinical factor (durable benefit or immunotherapy-related toxicity). The optimistic prognosis for these majority of patients should reassure both patients and providers in making the decision to stop immunotherapy.

## Supplementary Material

Supplemental Figure S1 KM curves for stopping immunotherapy due to disease control or toxicity
