# Supplementary figures and images for: Sustained Disease Control in Immune Checkpoint Blockade Responders with Microsatellite Instability-high Colorectal Cancer after Treatment Termination
Source: Cancer Res Commun. 2023 Dec 11;3(12):2510–7. doi: 10.1158/2767-9764.CRC-23-0340 (PMC10712284; doi:10.1158/2767-9764.CRC-23-0340)

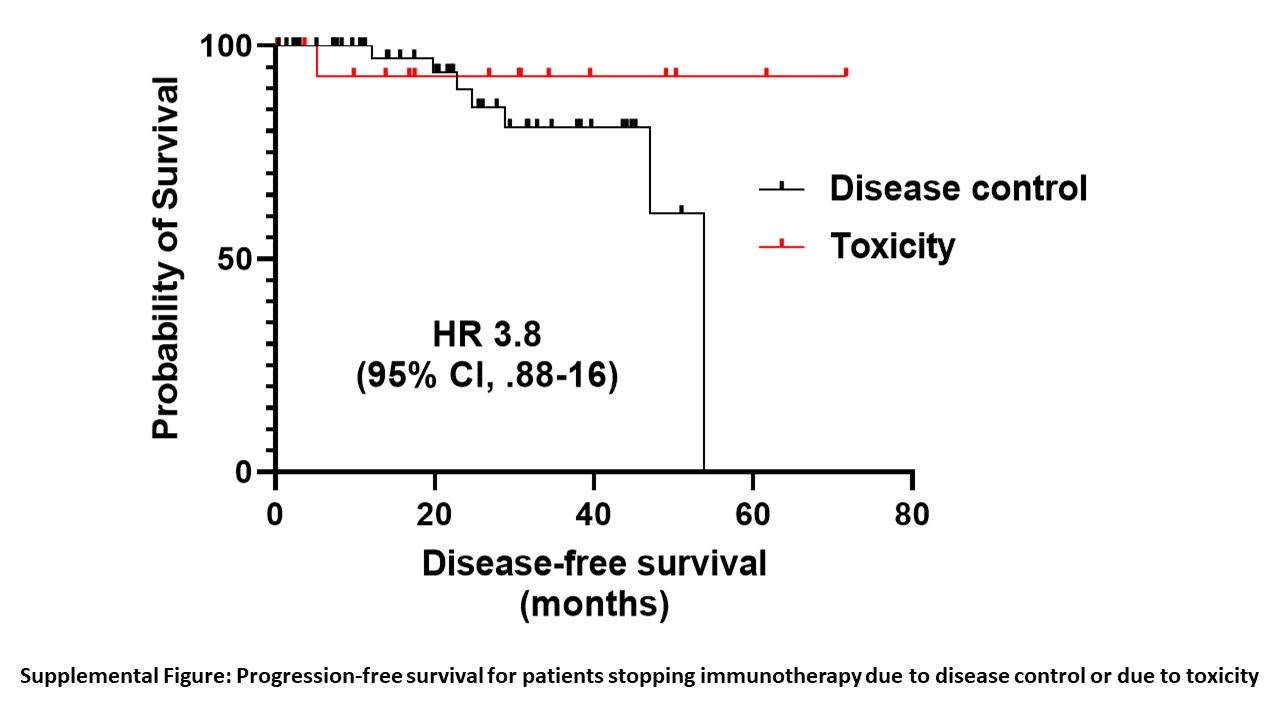

Supplement: Supplemental Figure S1 — KM curves for stopping immunotherapy due to disease control or toxicity [file crc-23-0340-s01.png]
